# A novel stability-indicating chromatographic quantification of the antiparkinsonian drug safinamide in its pharmaceutical formulation employing HPTLC densitometry and ion-pair HPLC–DAD

**DOI:** 10.1186/s13065-024-01315-y

**Published:** 2024-11-01

**Authors:** Engy A. Ibrahim, Samah S. Saad, Maha A. Hegazy, Laila E. Abdel Fattah, Hoda M. Marzouk

**Affiliations:** 1 Pharmaceutical Analytical Chemistry Department, College of Pharmaceutical Sciences and Drug Manufacturing, Misr University for Science & Technology, 6th of October City, Giza, Egypt; 2 Pharmaceutical Chemistry Department, Faculty of Pharmacy, Future University in Egypt, Cairo, 11835 Egypt; 3 Pharmaceutical Analytical Chemistry Department, Faculty of Pharmacy, Cairo University, Kasr Al-Aini Street, Cairo, 11562 Egypt

**Keywords:** HPTLC-densitometry, HPLC–DAD, In-vitro release, Safinamide Mesylate, Stability indicating methods, 4-hydroxy benzaldehyde, RGB12 model, Greenness assessment, Blueness assessment

## Abstract

Parkinson's disease (PD) emerges as a notable health concern among the elderly population. Safinamide mesylate (SAF) is a novel and emerging add-on therapy in PD treatment. The stability of innovative drug formulations and the development of appropriate stability-indicating methods are of great importance to modern pharmaceutical analysis. The current work has established novel comprehensive stability-indicating chromatographic approaches, HPTLC coupled with densitometric quantification and HPLC–DAD, for the selective assay of SAF in pharmaceutical formulation along with its synthetic precursor impurity; 4-hydroxy benzaldehyde (4-HBD) in presence of its stress induced degradation products. The stability of SAF was investigated under different stress conditions. It was found that SAF is likely to undergo acid, base hydrolysis, and oxidative degradation. Using mass spectrometry and infrared spectroscopy, the structures of the forced degradation products were confirmed and elucidated. The dissolution behavior of Parkimedine^®^ Tablets was also monitored in the FDA suitable medium. Multiple assessment tools were used to evaluate the environmental sustainability of the proposed methods and the reported one. The greenness tools included Complex-GAPI and AGREE metrics. In addition, the innovative concepts of "blueness" and "whiteness" evaluation were incorporated through the newly introduced BAGI and RGB12 algorithms, respectively.

**Supplementary Information:**

The online version contains supplementary material available at 10.1186/s13065-024-01315-y.

## Introduction

Neurodegenerative diseases have emerged as prevalent contributors to both mortality and morbidity worldwide, particularly affecting the elderly population. Parkinson's disease (PD) is the second most common neurological disorder globally [1]. Currently, no treatment is available to halt the progression of this disease, and the focus of management is primarily on alleviating motor symptoms. Dopamine-enhancing agents are typically used as first-line therapy [2]. Despite their efficacy in the early stages of the disease, there is a growing recognition of persistent symptoms and widely reported motor complications associated with long-term treatment [3]. MAO-B inhibitors have been proposed as a potential treatment for individuals with PD, as they help to prevent the breakdown of dopamine [4].

**Fig. 1:**
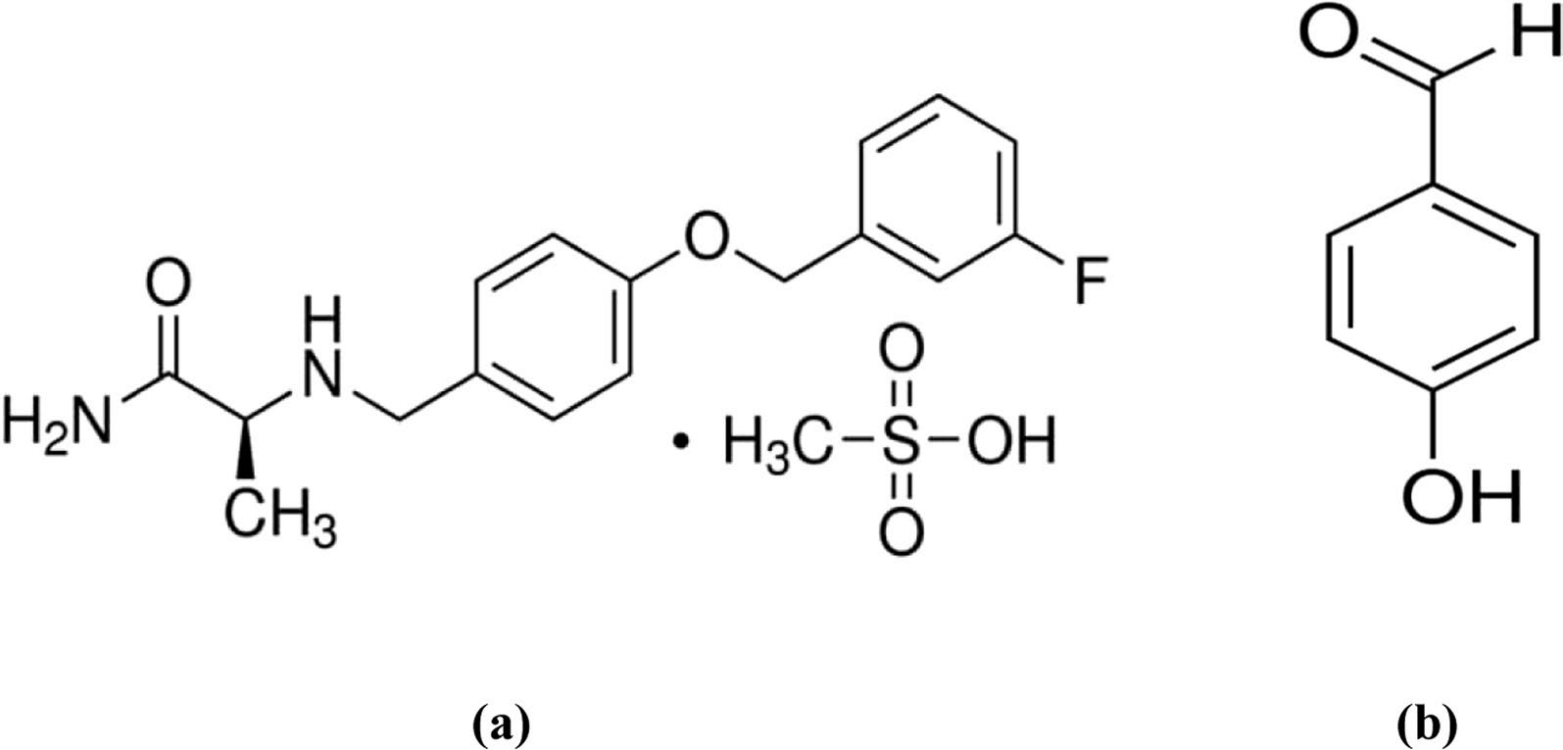
Chemical structure of **a** Safinamide Mesylate (SAF), and **b** 4-Hydroxybenzaldehyde (4-HBD)

Safinamide mesylate (SAF), chemically named (*S*)-2-[[4-[(3-fluorophenyl) methoxy]phenyl] methyl] aminopropanamide methane sulfonate [5, 6], is a novel drug that acts on dopaminergic and non-dopaminergic receptors [7]. It helps manage dopamine levels by inhibiting monoamine oxidase B (MAO-B) and reducing glutamate release [8]. The European Medicine Agency (EMA) and US-FDA approved it as an adjunctive treatment for PD patients in mid to late stages who are already taking levodopa alone or with other medications [7, 9]. *4*-Hydroxybenzaldehyde (4-HBD) is the synthetic precursor of SAF [10]. Figure 1 provides the chemical structure of SAF and its related substance.

The stability of new drugs is critical in examining their safety and efficacy. It assists in selecting the right formulations and packaging and provides an estimate of the ideal storage and shelf-life conditions. Subjecting new drug substances to conditions that are more harsh than accelerated conditions is employed to identify potential stress pathways and degradation products, aiding in the determination of the structure of these degradation products [11–13].

Chromatography is the most commonly used technique in pharmaceutical industry because it is cost-effective, easily accessible, sensitive, requires a small sample size, and its versatile applications [14–20]. Ion pair chromatography (IPC) is applicable in various analytical chemistry fields that utilize chromatography. It achieves effective separation by forming ion pairs between ionizable analytes and ion-pairing reagents with opposite charges. This method enhances compound separation, peak resolution, and consistently reproducible performance, resulting in improved peak shape [21, 22]. Our enhanced IPC technique, utilizing the readily accessible octane sulphonic acid sodium salt as an ion-pair reagent, allows for swift quantification in just 10.0 min. The brief analysis time provides the benefits of increased efficiency, reduced organic solvent consumption, and enhanced column durability, resulting in environmental friendliness and cost-effectiveness. Currently, there is a global acknowledgment of the significance of implementing green chemistry and employing its principles and tools. The analysts aimed to reduce or eliminate the risks associated with specific chemical substances, thus creating a more environmentally friendly laboratory environment [17, 23–25]. White analytical chemistry (WAC) is an extension of green analytical chemistry (GAC) [26], that incorporates green principles and other significant factors that impact analytical method quality. The WAC demonstrates the alignment and effectiveness of the analytical, ecological, and practical aspects [27].

A comprehensive literature analysis has revealed different analytical methods that can determine SAF alone or combined with other drugs, including HPLC [10, 28–32], UPLC [33], HPTLC [34], UV-spectrophotometric [35], and spectrofluorimetric method [36], as well as potentiometric and voltammetric methods [37, 38]. To the best of our knowledge, there is currently no stability-indicating method that is both eco-friendly and time-efficient for simultaneously determining SAF in presence of its stress induced degradation products as well as its synthetic precursor impurity.

The present study aims to build up and validate stability-indicating HPTLC-densitometric and HPLC–DAD methods for quantifying SAF in its pharmaceutical formulation along with its synthetic precursor impurity; 4-HBD in presence of its stress induced degradation products. The chemical stability of SAF was assessed across various stress conditions, including hydrolysis, oxidation, photolysis, and thermal stress conditions. The identification of the degradation products was elucidated by mass spectrometry (MS) and infrared (IR), and the postulation of degradation pathways was conducted. Furthermore, the HPLC–DAD method was utilized to track the dissolution profile of SAF formulated as Parkimedine® Tablets in FDA recommended medium. During the methods’ development, particular attention was given to the choice of solvent in order to enhance their sustainable and inexpensive characteristics. Moreover, this study thoroughly assesses the environmental impact of the proposed methods by utilizing multiple evaluation tools and compares them against the reported method [10]. In addition, we utilize the innovative notions of "blueness" and "whiteness" evaluation through the newly introduced BAGI and RGB12 algorithms.

## Experimental

### Equipment

#### For HPTLC-densitometric method

The chromatographic separation was developed using silica gel 60 F_254_ HPTLC plates (Merck, Darmstadt, Germany). A Camag Linomat-5 autosampler and Camag micro-syringe (Muttenz, Switzerland) were used for sample application. Densitometric scanning and measurements were conducted using the Camag TLC scanner (model number 3S/N 1302319) with the aid of winCATS® software. Reflectance-absorbance scanning was set at 20 mm/s with a slit measuring 3.0 × 0.45 mm. The peak areas of the obtained densitograms were recorded.

#### For HPLC–DAD method

The chromatographic procedure was conducted using an Agilent 1260 infinity series LC (Agilent, Santa Clara, California, USA), operated with a quaternary pump and a diode array detector. The samples were injected using an auto-sampler. The ChemStation 32 software system was used for data processing. The separation was performed using a Zorbax SB-C_18_ column (150.0 mm × 4.6 mm, 5.0 µm) (Agilent Technologies™, USA). A Jenway pH apparatus (model 3510, UK) was used for pH adjustments.

Mass scan was performed using Waters UPLC^®^ system (Waters Co., USA) equipped with Waters Acquity Triple-Quad detector (MS/MS), binary solvent-delivering system and an autosampler. The system employs positive electrospray ionization (ESI) mode for the structure elucidation of intact SAF and its corresponding degradation products. The data were obtained and integrated using Mass Lynx 4.1 SCN805 software.

IR spectra were collected using a Shimadzu IR Affinity-1 FT-IR Spectrophotometer (Kyoto, Japan), accompanied by lab solution software.

#### Dissolution test apparatus

The dissolution profiling was conducted using the Vision^®^ G2 Elite 8™ instrument (Hanson, USA), outfitted with a standard USP type-II paddle and eight vessels.

### Chemicals and reagents

Pure SAF (certified to be 100.70%) was supplied by EVA Pharma for Pharmaceutical Industry (Al-Giza, Egypt). 4-HBD standard (purity, 99%) was provided by Acros Organics, Fisher Scientific (Belgium).

Parkimedine® Tablets, manufactured by EVA Pharma for the Pharmaceutical Industry (B.N. (10) 2112425), are formulated to contain 100 mg of SAF per tablet.

Methanol used in this study was HPLC-grade and provided by Merck (Darmstadt, Germany). Also, ethanol HPLC-grade was supplied from Fisher scientific (Germany). 1-Octane sulphonic acid sodium salt was obtained from CDH Chemicals (Delhi, India). Analytical-grade solvents and reagents were utilized in the study. ortho-Phosphoric acid was sourced from El-Nasr Pharmaceutical Chemicals Co. (Cairo, Egypt). Ethyl acetate, aqueous ammonium hydroxide solution (25%), hydrochloric acid (37%), sodium hydroxide, and hydrogen peroxide (30%) were obtained from PioChem Co. (Giza, Egypt). Sodium dihydrogen phosphate was procured from Advent Chembio Private Ltd. (India). Otsuka Pharmaceutical Co. (Cairo, Egypt) supplied the deionized distilled water used in the study.

### Chromatographic conditions

#### For HPTLC-densitometric method

In the HPTLC method, the stationary phase; is typically a thin silica gel 60 F_254_ layer coated onto an aluminium sheet measuring 20.0 × 10.0 cm for chromatographic separation. Samples to be analyzed are applied on the plate in triplicates, and the plate is placed into a developing chamber filled with a solvent mixture of ethyl acetate—methanol—aqueous ammonium hydroxide solution (9.0: 1.2: 0.1, by volume). The applied samples were in bands 6.0 mm wide, positioned 10.0 mm away from the sides and bottom edge of the plates, using a Camag autosampler. Before ascending development, the chamber was left to saturate at room temperature for 30 min. The development was conducted over 8.0 cm. The developed plates were taken out and allowed to dry in the air. After drying, they are scanned at a wavelength of 210.0 nm using the same instrumental conditions mentioned before.

#### For HPLC–DAD method

Separation of SAF, its degradation products, and 4-HBD was accomplished using an isocratic elution on a Zorbax SB-C_18_ column (150.0 mm × 4.6 mm, 5.0 µm) maintained at 40 ^◦^C. The mobile phase consisted of a mixture of 25.0 mM sodium dihydrogen phosphate containing 0.1% (w/v) octane sulfonic acid sodium salt adjusted with o-phosphoric acid to pH 5.0 ± 0.1 and methanol (45:55, v/v). The flow rate was set to 1.2 mL/min and adjusted to 1.5 mL/min from 3.1 min onward. Before use, the mobile phase underwent filtration through a 0.45  µm membrane filter then degassed by ultra-sonication for 15.0 min. Each sample was filtered and then injected in triplicates via autosampler into the LC system with 10.0 µL injection volume. The separation run time was accomplished in 10.0 min, and DAD scanning was carried out at 225.0 nm.

### Stock and working standard solutions

Stock standard solutions of SAF, 4-HBD, and SAF degradation products were separately prepared in methanol at a concentration of 1.0 mg/mL. The HPTLC method used the stock solutions without any changes, while the HPLC procedure needed working standard solutions (100.0 μg/mL) prepared by diluting the standards with the mobile phase.

### Linearity and construction of calibration curve

#### For HPTLC-densitometric method

The volumes were applied as separate bands in triplicates from their respective stock solutions using the HPTLC system, following the chromatographic conditions that were previously described. The concentration range was 2.00–15.0 and 0.50–3.40 µg/band for SAF and 4-HBD, respectively. The scanning profiles have been obtained, and the calibration plots have been created to determine the correlation between the concentration of each analyte and the average peak area. The polynomial regression equations have also been calculated.

#### For HPLC–DAD method

Measured volumes of each analyte were accurately transferred from their respective stock and working standard solutions into a 10-mL measuring flask and then diluted with the mobile phase. The solutions were prepared in the concentration range of 3.00–150.0 and 0.50–7.00 μg/mL for SAF and 4-HBD, respectively. The samples were prepared in mixtures and injected into the HPLC–DAD system three times using the pre-specified chromatographic conditions. Calibration curves were constructed by plotting the average peak areas against the corresponding drug concentrations.

### Stress stability studies

Stability studies were performed under acid, base hydrolysis, oxidative, photolytic, and thermal stress conditions following the ICH recommendations for stability testing of new drug substances and products [39].

Acid and basic hydrolysis were examined by transferring an exact volume of 20.0 mL from SAF stock standard solution (1.0 mg/mL) into separate round flasks containing 30.0 mL of different aqueous hydrochloric acid or methanolic sodium hydroxide (2–5 N). The prepared round flasks were refluxed at 100 ^◦^C for 5 h. Samples were treated using equal strength of either aqueous sodium hydroxide or hydrochloric acid for neutralization. Samples evaporation was performed until dryness, washed twice with 10.0 mL methanol, and filtered.

For oxidative stress investigation, an exact volume of 20.0 mL from SAF stock standard solution (1.0 mg/mL) was placed into separate round flasks containing 30.0 mL different strengths of aqueous hydrogen peroxide solutions (10, 15, 30%) and left for 24 h at room temperature in a dark place. Then, the solutions were evaporated completely and re-dissolved in methanol.

Photo-stability of the SAF solution (10.0 µg/mL) was examined through a 6 h exposure to direct sunlight.

Finally, to study the impact of dry heat, SAF was subjected to thermal stress testing in the solid-state by being kept in a thermostatically controlled oven at 110 °C for 5 h. In addition, SAF aqueous solution was refluxed at 350 °C for 4 h to study thermal stress.

The degradation process was tracked, samples were taken at regular intervals, diluted appropriately with methanol, and then analyzed using the previously discussed HPTLC-densitometric methods.

The isolated degradation products were subjected to IR and MS for identification and confirmation.

### Analysis of pharmaceutical formulation

Ten Parkimedine^®^ Tablets (100 mg SAF per tablet) were subjected to individual weighing, followed by powdering and thorough mixing in a hand mortar. Subsequently, an accurately measured amount equivalent to the weight of a one tablet was transferred into a 50-mL volumetric flask and sonicated for 30 min with 30.0 mL methanol. Then, the volume was completed with methanol to the mark and filtered through a 0.45 μm nylon sample filter. The previously prepared solution underwent further dilution with methanol for HPTLC or mobile phase for HPLC, aiming to achieve concentrations that fall within the SAF linearity ranges. The procedures were then performed following the instructions supported by each method. A regression equation was used to determine the concentration of SAF in tablets.

### Application to in-vitro dissolution studies

The dissolution behavior of Parkimedine^®^ Tablets in water was monitored using the HPLC–DAD procedure, following the FDA dissolution method [40]. The dissolution test was conducted using a USP dissolution apparatus II, where a single tablet was introduced into a vessel containing 900 mL 0.1 N hydrochloric acid with sodium chloride (0.2% w/v), pH 1.2, as the dissolution medium. The system was then equilibrated at 37 ± 0.5 °C and stirred at 100 rpm. Samples of 5.0 mL were taken out at pre-determined time intervals of 5, 10, 15, 30, 45, and 60 min, and the collected volume was replaced with an equivalent volume of fresh medium. After the collection, the samples were filtrated and injected, in triplicates, into the HPLC system. The in-vitro release profile of SAF was monitored. The resulting dissolution percentage was determined by utilizing the appropriate regression equation for SAF in the stated medium.

## Results and discussion

### Degradation behavior of SAF under various stress conditions

As per the ICH guidelines [39], it is necessary to conduct individual studies that investigate the degradation pattern under various stress conditions, including acid, alkali hydrolysis, oxidation, exposure to light, and dry/wet heat. The proposed HPTLC method was used for tracking the degradation process.

**Fig. 2:**
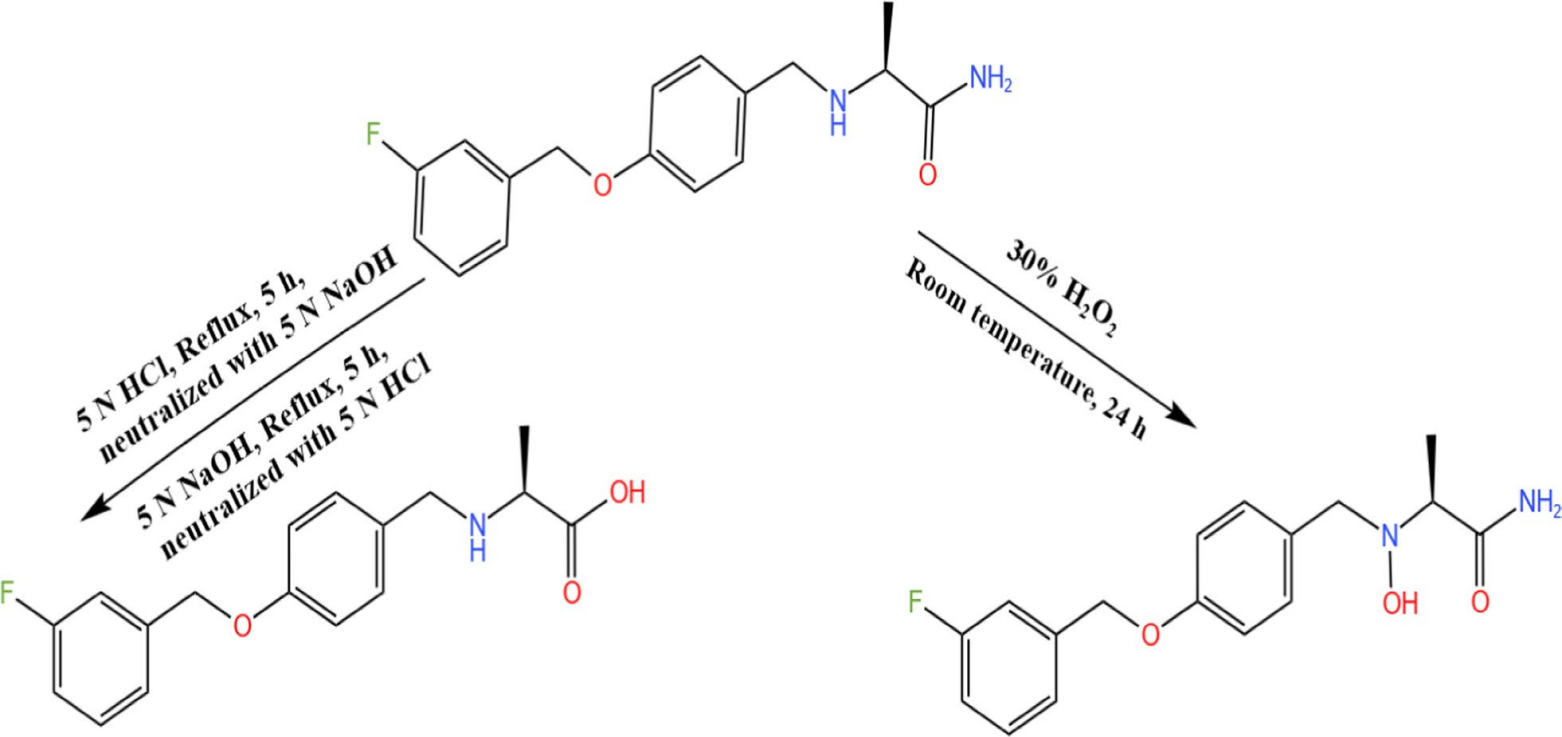
The suggested pathways for safinamide-induced degradation

It was established that SAF undergoes complete degradation after 5 h at 100 °C in the presence of either aqueous 5 N HCl or methanolic 5 N NaOH. This was proven by the disappearance of the SAF band and the appearance of a new degradation one instead, as shown in Fig. S1. SAF hydrolysis under acid and alkali conditions was achieved through the amide group breaking down, releasing the free amine and the corresponding acid, according to the proposed pathway presented in Fig. 2.

The SAF oxidation process involved leaving it to stand with 30% H_2_O_2_ at room temperature for 24 h. This was confirmed by the disappearance of the SAF intact band and appearance of new degradation one, as presented in Fig. S1. The reaction is proposed to occur by secondary amine oxidation using hydrogen peroxide, which donates oxygen to nitrogen, as shown in Fig. 2.

SAF showed relative stability under the direct sunlight exposure for 6 h and exhibit dry and wet heat stability with no degradation signs under previously mentioned thermal stress conditions, where the densitograms for both conditions only showed the intact SAF band with no other bands, as illustrated in Fig. S1.

The intact drug and SAF induced degradation products were subjected to structural elucidation using IR and MS. The IR and MS spectra of the degradants were compared with that of the intact drug. The comparison of the IR spectra reveals that the characteristic forked N–H bands at ∼3300 and 3350 cm^−1^ have disappeared, as presented in Fig. S2a. In contrast, a new broad O–H band at ∼3450 cm^−1^ has appeared, as shown in Fig. S2b, c. The IR spectrum for the oxidative degradation product shows a broad O–H band and forked N–H bands in the region of 3100–3500 cm^−1^, as illustrated in Fig. S2d. Positive electrospray ionization was utilized to compare the mass spectral data. The acid and basic degradation products had molecular ion peaks at m/z 304. The SAF oxidative degradation product had a molecular ion peak at m/z 318, as shown in Fig S3.

### Method development and optimization

#### For HPTLC-densitometry

**Fig. 3:**
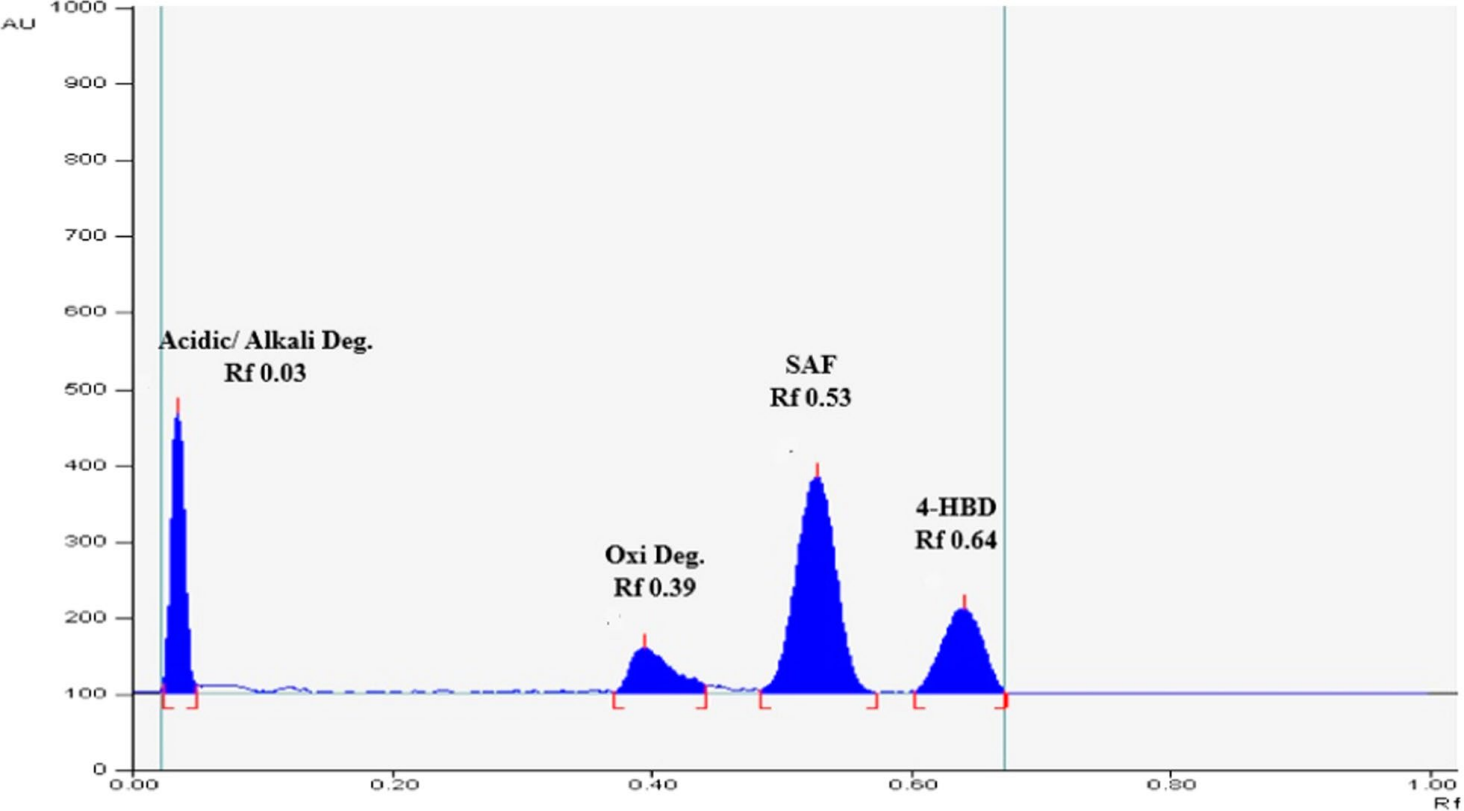
HPTLC-densitogram of a resolved mixture of SAF (2.0 µg/band), 4-HBD (0.5 µg/band) and SAF pooled degradation products

Multiple experimental trials were conducted to establish the most favorable chromatographic conditions for accurately quantifying SAF in the presence of its degradation products and precursor impurity. Various eco-friendly developing systems were tested with different ratios to identify the most suitable one. The trials started by using ethyl acetate alone, ethyl acetate combined with ethanol or methanol in varying ratios (6:4, 7:3, 8:2, 9:1, 9.5:0.5, 9.5:1, and 9:1.2, v/v). However, the results were unsatisfactory with significant tailing, especially for degradation products along with incomplete separation of the studied compounds. Subsequently, varying quantities of aqueous ammonium hydroxide (0.05–0.15 mL) and glacial acetic acid (0.05–0.1 mL) were investigated as potential additives. Preliminary results indicated that the inclusion of aqueous ammonium hydroxide yielded favorable outcomes at the volume of 0.1 mL, exhibiting improved peak shapes and satisfactory resolution. The increased levels of more than 0.1 mL aqueous ammonium hydroxide have resulted in the migration of 4-HBD towards the solvent front. Glacial acetic acid usage retained the acid and basic induced degradation products on the baseline. Finally, the combination of ethyl acetate, methanol and aqueous ammonium hydroxide (9:1.2:0.1, by volume) as the developing system produced a successful separation with suitable R_f_ values and minimal tailing, as depicted in Fig. 3. The densitometric measurements involved the evaluation of different scanning wavelengths (210.0, 225.0, 254.0, and 280.0 nm), considering the absorbance spectra of the compounds under investigation. The best sensitivity and peak symmetry were obtained by scanning at 210.0 nm, as shown in Fig. 3. The HPTLC method used the following elution sequence: The acidic/alkali degradation product has a carboxylic acid group that can create potent hydrogen bonds with silica gel. Due to its strong interaction with the stationary phase, it is the most polar compound, showing an Rf value (0.03). The oxidative degradation product has an additional hydroxy amino group compared to SAF, which makes it more polar. The hydroxy amino group forms hydrogen bonds with the stationary phase, leading to a more significant reduction in Rf (0.39) compared to SAF. SAF exhibits a moderate level of polarity due to amide and amine groups. However, it is still less polar compared to the first two compounds. 4-HBD has a higher Rf (0.64) due to its moderate polarity and stronger affinity for the relatively polar mobile phase, allowing it to migrate further along the TLC plate. To evaluate the suitability of the chromatographic system, different parameters are calculated for each of the four components [41]. Table 1 displays that all the calculated criteria fell within the approved ranges.

**Table 1:** System suitability parameters of the proposed HPTLC-densitometric and HPLC–DAD methods

Method	Parameter	Acid/Alk.Deg	Oxi. Deg	SAF	4-HBD	Reference value [41]
HPTLC-densitometry	Retardation factor (Rf) ± 0.02^a^	0.03	0.39	0.53	0.64	
	Capacity factor (k’)^b^	32.33	1.56	0.88	0.56	
	Selectivity (α)^c^	20.7 1.77 1.57				> 1
	Resolution (Rs)^d^	7.52 1.94 1.82				Rs > 1.5
	Tailing factor (T)	1.00	1.50	1.00	1.00	T ≤ 2
HPLC–DAD	Parameter	4-HBD	Oxi Deg	Acid/Alk Deg	SAF	Reference value [42]
	Retention time (min ± 0.2)	1.91	2.41	4.07	5.63	
	Selectivity (α)^c^	1.71 2.37 1.54				> 1
	Resolution (Rs)^d^	1.72 4.28 4.14				Rs > 1.5
	Tailing factor (T)	1.00	1.33	1.00	1.00	T ≤ 2
	Column efficiency (N)	5836	1486	2946	3167	N ˃ 2000
	Height equivalent to theoretical plate HETP (cm/plate)	2.57 × 10^–3^	1.00 × 10^–2^	5.09 × 10^–3^	4.74 × 10^–3^	

^a^Retardation factor (*R*_*f*_) = distance travelled by the analyte/distance travelled by the solvent front

^b^Capacity factor (*k’*) = (1-*R*_*f*_)/*R*_f_

^c^Selectivity (α) = *k’*_2_/*k’*_1_ calculated for each of two successive peaks

^d^Resolution (R_s_) = *Rf*_*2*_–*Rf*_*1*_/0.5 (w_1_ + w_2_), where *R*_*f*_ is the retardation factor, and w is the peak width calculated for each of two successive peaks for HPTLC and = 2(t_RB_–t_RA)/(_W_B+_W_A_) for HPLC

#### For HPLC–DAD method

Separating SAF and its stress-induced degradation products simultaneously presented a challenge due to their relative structural similarity. A new method has been developed for the quantitative analysis of SAF and 4-HBD in presence of SAF degradation products together with high resolution. This approach aims to offer a reliable, user-friendly, sustainable, and effective solution. Different ratios of deionized water and 25.0 mM sodium dihydrogen phosphate buffer were utilized as the aqueous phase, and ethanol and methanol were used as the organic phase. The ratios tested were 30:70, 40:60, 50:50, and 45:55, v/v, with pH levels ranging from 4.0 to 6.0 ± 0.1. Additionally, flow rates ranging from 1.0 to 1.5 were tested. However, poor resolution and increased column pressure were observed when ethanol was used as the organic modifier. In contrast, using methanol as an organic modifier resulted in high resolution and good peak symmetry. Incomplete separation was observed in different ratios when deionized water was used as the aqueous phase. The separation was enhanced for the four compounds while using the phosphate buffer at pH 5.0 ± 0.1, but the peaks showed tailing and delaying in the run time for 15 min. In order to enhance the symmetry and resolution of the peaks, 0.1% (w/v) octane sulphonic acid sodium salt was added to the phosphate buffer as an ion-pairing reagent. The sulfonate ions (SO_3_^−^) can engage in hydrogen bonding, enabling them to establish electrostatic interactions with analytes that carry opposite charges. To achieve the best resolution for SAF, 4-HBD and its stress degradation products, an isocratic elution was used with a 25.0 mM sodium dihydrogen phosphate buffer containing 0.1% (w/v) octane sulfonic acid sodium salt adjusted with *o*-phosphoric acid to pH 5.0 ± 0.1 and methanol (45:55, v/v). The implementation of flow rate programming has been attempted. By adjusting the flow rate to 1.2 mL/min and setting it to 1.5 mL/min at 3.1 min, the observed outcome was the generation of sharp peaks, leading to a decrease in analysis time.

Various columns, including Zorbax SB-C_8_ (150.0 × 4.6 mm, 5.0 µm; Agilent Technologies, USA), Zorbax SB-C_18_ (75.0 mm × 4.6 mm, 3.5 µm; Agilent Technologies, USA), Zorbax SB-C_18_ (150.0 × 4.6 mm, 5.0 µm; Agilent Technologies, USA), and Hypersil C_18_ (250.0 × 4.6 mm, 5.0 μm; Thermo Scientific, USA), were used during the methodology development process. The use of the C_18_ column instead of C_8_ improved the separation process and provided optimal resolution for the analytes. Finally, Zorbax C_18_ (150.0 × 4.6 mm, 5.0 μm) provided exceptional resolution, producing sharp and distinct peaks.

**Fig. 4:**
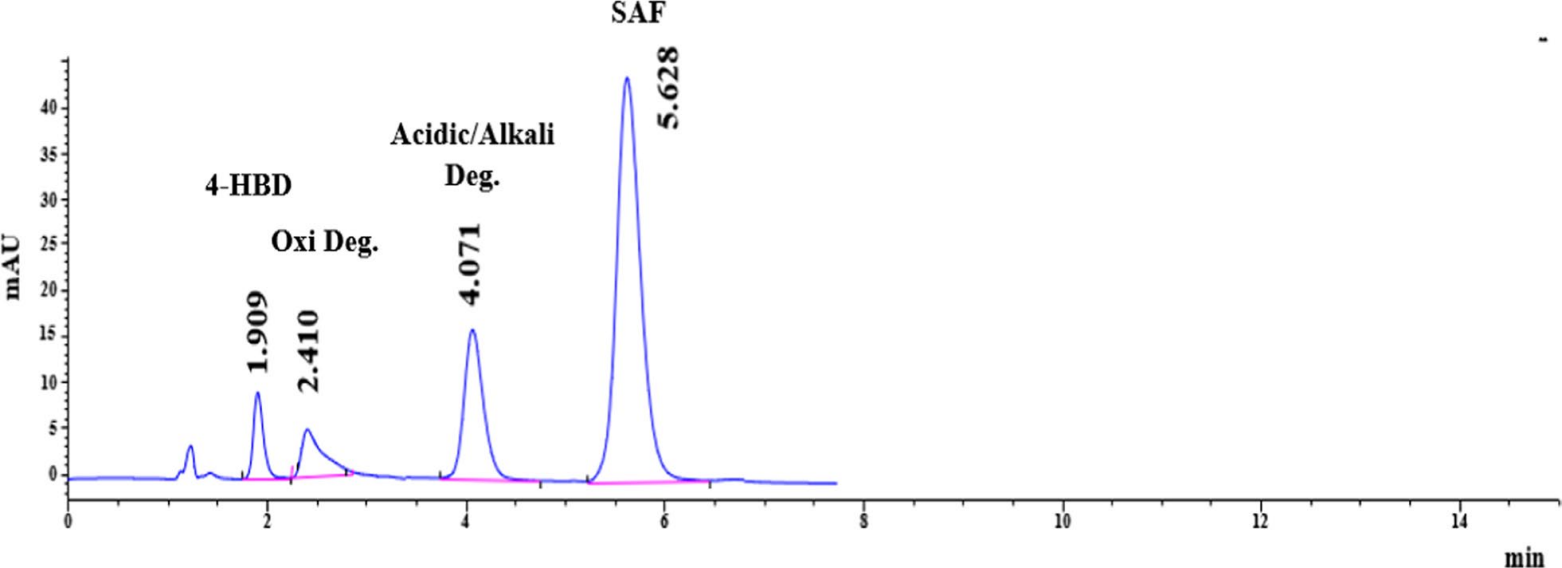
HPLC–DAD chromatogram of a resolved mixture of SAF (30.0 µg/mL), 4-HBD (1.00 µg/mL) along with SAF pooled degradation products

In addition, we explored the impact of column temperatures ranging from 25 to 40 ^◦^C. We found that using higher column temperatures resulted in a shorter analysis time due to decreased mobile phase viscosity, which improved the overall outcome. After careful consideration, we determined that the optimal temperature for proper peak resolution was 40 ^◦^C, as shown in Fig. 4.

In order to determine the best sensitivity and peak shape for the studied analyte, several detection wavelengths, including 210.0, 225.0, 230.0, and 254.0 nm, were tested. Based on the findings, the ideal wavelength for SAF determination was 225.0 nm, as shown in Fig. 4.

At pH 5.0, both 4-HBD and oxidative-degradation products appear neutral. Acid/alkali degradation products exhibit a zwitterionic structure, whereas SAF carries a positive charge. The ion-pairing agent used in the mobile phase was the octane sulfonic acid sodium salt, which carries a negative charge. The ion-exchange model relies on the interaction between the hydrophobic part of the ion-pair reagent and the hydrophobic stationary phase, resulting in the formation of a dynamic ion-exchange surface. Consequently, the proposed HPLC method showed the following elution order: the neutral compounds were eluted first, then the Zwitterionic compound. The last eluted component is SAF, which carries a positive charge.

Table 1 displays the results of calculating system suitability parameters to ensure the chromatographic system can effectively separate the intended substances. The parameters assessed include resolution, tailing factor, selectivity factor, column efficiency, and theoretical plates. These parameters meet the acceptance criteria [42], indicating that the method proposed is highly selective and successfully separates the peaks with a clear baseline.

### Evaluation of analytical method greenness

Green chemistry, or sustainable chemistry, aims to reduce or remove the harmful impact of chemicals on the environment [17, 43]. Implementing green analytical methods can help industries improve their environmental sustainability. Different ways have been created to measure how much the environment is affected by analytical methods. In this study, we used two tools, Complex-GAPI and AGREE, to evaluate how environmentally friendly these methods are.

#### Complementary green analytical procedure index (Complex-GAPI)

It is an update to the commonly used GAPI metric [44]. This assessment uses a visual representation to assess the methodology. The system uses five pentagrams, each representing a different aspect of methodology: sample collection and preparation, reagents and solvents, instrumentation, and method type. The pentagrams act as indicators for the overall quality and effectiveness of each aspect. The pre-analysis procedures are represented by a hexagonal field, in addition to the pentagrams. The inclusion of this field is important because the pre-analysis stage is a critical part of the overall methodology and can greatly affect the accuracy and reliability of the results. The color scheme used in the assessment is worth mentioning. It uses various colors to represent the environmental impact of each stage. Red is chosen to indicate environmentally hazardous steps, serving as a warning for potential risks. Yellow indicates actions with a moderate environmental impact, indicating a moderate level of concern. Green emphasizes eco-friendly actions, indicating that they are environmentally friendly and have minimal negative impacts. The assessment utilizes a visual representation and color scheme to provide a comprehensive and easily clear evaluation of the methodology, specifically focusing on its environmental impact and overall sustainability. The suggested approaches showed good greenness with 4 green shades and fewer red shades than the reported one [10] (Table 2). The hexagonal form appears white due to our tendency to prioritize instant measurement and the absence of pre-analysis planning. Table S1 illustrates the detailed differences between the proposed and reported methods.

**Table 2:** Greenness assessment of the proposed chromatographic methods and the reported one according to Complex-GAPI, AGREE tools, blueness assessment by BAGI tool and whiteness assessment by RGB 12 model

Method	Complex-GAPI assessment^a^	AGREE assessment^b^	BAGI^c^	RGB12^d^
Proposed HPTLC-densitometry Method				
Proposed HPLC–DAD Method				
Reported HPLC–UV Method [10]				

^a^Complex-GAPI Assessment is an update to the commonly used GAPI metric and evaluated according to Green Analytical Procedure Index parameters description [44]

^b^AGREE Assessment evaluated by using Analytical GREEnness Metric approach and Software [45]

^c^Blue Applicability Grade Index (BAGI) is proposed as a new metric tool for evaluating the practicality of an analytical method [46]

^d^RGB12 algorithm for whiteness evaluation is a unique ideology tool for applying sustainable development concepts in analytical chemistry [47]

#### Greenness assessment applying analytical GREEness metric (AGREE) approach

There are many techniques for estimating the greenness of analytical methods, but only AGREE software applies all 12 GAC principles to assess the analytical methodology's greenness [45]. As a result, AGREE: The Analytical Greenness Calculator was used to evaluate the method's greenness. Table 2 displays resulted pictograms/scores for the proposed chromatographic methods and the reported one [10]. According to the AGREE score calculations, the proposed methods are greener than the reported method, reaching 0.72, 0.68 and 0.62 for the proposed HPTLC, HPLC and reported methods, respectively. Table S2 showed the specific differences between the proposed and reported methods.

### Blue applicability grade index (BAGI)

The BAGI tool is a novel metric used to evaluate the practicality of an analytical method. BAGI focuses primarily on the utilization of White Analytical Chemistry and can be considered as a complementary addition to the already established green metrics [46]. The BAGI metric tool provides two distinct sets of results: an asteroid pictogram as a visual representation and a numerical score displayed at the center. The asteroid pictogram utilizes a range of blue hues to depict different levels of compliance, with dark blue indicating high compliance, blue indicating moderate compliance, light blue indicating low compliance, and white indicating non-compliance. This pictogram serves as a visual representation of the assessment result. The proposed methods demonstrated their superiority over the previously reported method in terms of time efficiency. As shown in Table 2, the suggested methods have a high BAGI score over the reported one [10], which attests to their good applicability.

### Whiteness tool for assessing analytical chemistry method

The WAC metric tool [27] was created to evaluate efficiency, environmental impact, and economic feasibility of analytical methods. Various sustainability assessment methods exist, each with its advantages and disadvantages. However, combining different methods consistently yields favourable outcomes. The Red–Green–Blue (RGB) model is helpful for evaluating analytical procedures worldwide. It uses a quantitative approach to evaluate how well the analytical approach promotes sustainability [47–49]. The combination of the primary colors red, green, and blue serves as the fundamental method for attaining a white outcome in the sustainability analysis of a product. The red component of the analytical method signifies its efficiency, which includes various factors such as the scope of application, limits of detection and quantification, precision, and accuracy. The GAC 12 principles are linked to the green color in sustainability analysis. These principles encompass various factors, including reagent toxicity, number of reagents, waste generation, energy consumption, and direct impacts. The analysis includes evaluating the blue component, which encompasses cost, time efficiency, requirements, and operational simplicity. A comprehensive excel worksheet has been prepared to assess the sustainability of the proposed analytical methods and the reported one [10] using the WAC metric. The worksheet includes a table with three columns representing red, green, and blue colors. The analysis yields a numerical score ranging from 0 to 100, which indicates the analytical method's overall sustainability. According to Table 2, it can be observed that the HPTLC-densitometry method exhibits a higher level of environmental friendliness when compared to the proposed HPLC–DAD method. The detailed difference between the proposed methods and the reported ones is illustrated in Table S3.

### Method validation

Parameters for method validation were evaluated in accordance with ICH guidelines [50] to guarantee the reliability of the proposed methods.

The correlation between the mean peak area and the associated concentration of each analyte was built into the calibration curves. Table 3 summarizes the linearity range and computed regression parameters for each analyte in the two proposed methods.

**Table 3:** Regression and validation parameters of the proposed HPTLC-densitometric and HPLC–UV methods for the determination of Safinamide along with its impurity in pure form

Method Parameter	HPTLC-densitometric method		HPLC–DAD method	
	SAF	4-HBD	SAF	4-HBD
Range	2.00–15.00 µg/band	0.50–3.40 µg/band	3.00–150.00 μg/mL	0.50–7.00 μg/mL
Regression equations parameters				
Slope (b)^a^	–	–	15.441	44.689
Coefficient 1 (b1)^b^	− 64.966	− 913.12	–	–
Coefficient 2 (b2)^b^	2317.3	6280	–	–
Intercept (a)^a, b^	324.49	− 1942.8	− 1.9547	8.0267
Correlation Coefficient (r)	0.9999	0.9999	1.000	0.9997
Accuracy (Mean ± SD)	100.13 ± 1.286	100.11 ± 1.389	100.24 ± 1.092	100.24 ± 1.631
Precision				
(%RSD)^c^	0.291	0.674	0.309	0.656
(%RSD)^d^	1.252	0.842	0.745	1.495
LOD^e^	–	0.019	–	0.153
LOQ^e^	–	0.056	–	0.463
Robustness^f^	1.658	1.944	1.952	1.913

^a^Regression equation for HPLC: *A* = *a* + *bc*, where ‘A’ is the average peak area and ‘c’ is the concentration (μg/mL)

^b^Coefficient 1 and 2 are the coefficients of X^2^ and X, respectively. Following a polynomial regression: *A* = *b1x*^*2*^ + *b2x* + *a*, where ‘A’ is the average peak area, ‘c’ is the concentration (μg/band), ‘b1’ and ‘b2’ are coefficients 1 and 2, respectively and ‘a’ is the intercept

^c^Intra-day precision [average of three different concentration of three replicates each (n = 9) within the same day], for HPTLC the concentrations were (4.00, 6.00, 10.00 µg/band) for SAF, and (1.00, 1.40, 2.40 µg/band) for 4-HBD, For HPLC: the concentrations were (0.80, 3.00, 5.00 μg/mL) for 4-HBD, and (44.00, 66.00, 132.00 μg/mL) for SAF

^d^Inter-day precision [average of three different concentration of three replicates each (n = 9) repeated on three successive days], the concentrations were the same as in intra-day precision

^e^LOD and LOQ are calculated according to ICH, 3.3 × SD of the residuals/slope and 10 × SD of the residuals/slope, respectively

^f^For HPTLC: average of the change in scanning wavelength (±1 nm), ethyl acetate ratio (±1%), developing distance (±0.5 cm) and saturation time (±5 min). For HPLC: average for flow rate (±0.1 mL/min) and pH (±0.1)

To determine the accuracy of the proposed methods, two pure samples of SAF and 4-HBD were analyzed using the previous chromatographic conditions. Five different concentrations of each were calculated using the regression equation. The mean %recoveries confirming the accuracy of the suggested methods were satisfactory, as shown in Table 3.

We conducted triplicate analyses of three chosen concentrations for both drugs to gauge the method's precision. To assess intra-day precision, these analyses were conducted on the same day, and to evaluate inter-day precision, they were conducted over three days in consecutive days. Table 3 shows the resulting relative standard deviations (RSD%) were under 2%.

The effective separation of the investigated drug, along with its impurity and degradation products, serves as evidence of the selectivity of the proposed methods, as depicted in Figs. 3 and 4. Furthermore, the high % recoveries of SAF in its pharmaceutical formulation guarantee the absence of chromatographic interference caused by commonly used tablet excipients, as indicated in Table 4. The level of specificity was ensured by utilizing the winCATS spectral correlation tool for HPTLC or online monitoring by DAD for HPLC. This allowed for the assessment of purity for each drug peak during elution.

**Table 4:** Results obtained by applying the proposed HPTLC-densitometric and HPLC–DAD methods for the determination of Safinamide in Parkimedine^®^ Tablets and application of standard addition technique

Pharmaceutical formulation		HPTLC-densitometry					HPLC–DAD method				
				Standard Addition Technique					Standard Addition Technique		
Parkimedine^®^ Tablets		Drug	%Found ± SD^a^	Claimed (µg/band)	Pure added (µg/band)	%Recovery of the pure added^b^	Drug	%Found ± SD^a^	Claimed (μg/mL)	Pure added (μg/mL)	%Recovery of the pure added^b^
(Each tablet is labelled to contain 100 mg SAF)		**SAF**	100.13 ± 1.815	4	2.00	100.15	**SAF**	99.63 ± 0.523	20	10.00	99.23
					4.00	101.15				20.00	100.34
					8.00	100.28				40.00	99.60
				**Mean ± SD**		100.52 ± 0.549			**Mean ± SD**		99.72 ± 0.564

^a^Average of five determinations

^b^Average of three experiments

The limits of detection (LOD) and limits of quantification (LOQ) were determined for 4-HBD by utilizing the slope of the standard calibration curve and the standard deviation of the residuals. The LOD and LOQ values obtained demonstrate the high sensitivity of the proposed methods, as shown in Table 3.

The robustness of the developed methods was evaluated by determining the RSD% after slightly modifying the optimized experimental parameters. The system suitability parameters exhibited no significant influence, and the % RSD values were less than 2%, as presented in Table 3.

### Analysis of the dosage form (Parkimedine^®^ Tablets)

The validated methods efficiently determined SAF in its pharmaceutical formulation, Parkimedine^®^ Tablets. The sample preparation process involved a single extraction step using methanol. This extraction method proved to be effective in extracting SAF from the tablet matrix, ensuring that there were no interferences from the tablet excipients. This is crucial in obtaining accurate results and ensuring the specificity of the analysis. The validity of the methods was confirmed through the standard addition technique, as shown in Table 4. The favorable outcomes and the limited number of steps involved in sample manipulation emphasize the practicality and environmentally friendly nature of these proposed methods for routine quality control of the specified medication.

### Dissolution testing of Parkimedine^®^ tablets by the proposed HPLC method

**Fig. 5:**
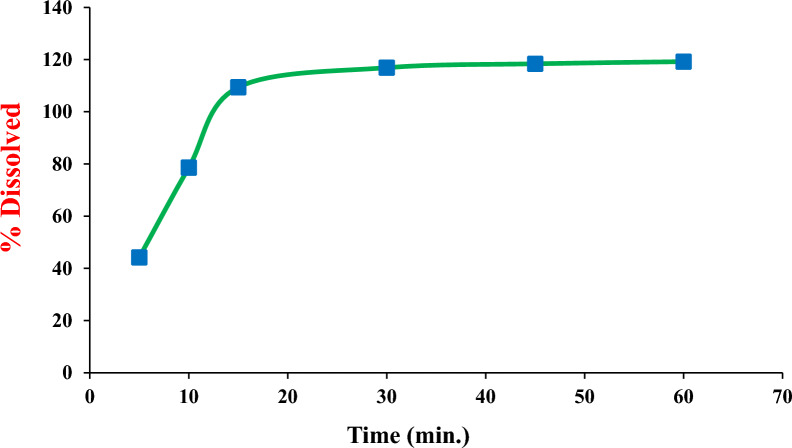
Dissolution profile of Parkimedine^®^ Tablets in 0.1 N HCl as FDA-dissolution medium using the developed HPLC–DAD method

It is essential to establish an in-vitro dissolution profile as part of the drug development process. This is a vital process to establish a link between in-vitro and in-vivo behaviour and to guarantee uniformity between batches. Based on the FDA dissolution methods database, the cited drug's in-vitro release was monitored in 0.1 N HCl with a pH of 1.2 as the dissolution medium. The drug release profile was constructed by plotting the percentage of dissolved drug against time, as shown in Fig. 5. The current study found that over 75% of the studied drug was released from Parkimedine^®^ Tablets in 0.1 N HCl with a pH of 1.2 within 10 min. This result indicates that the specified acceptance criteria, which involve the quantity of active substance dissolved within a specific time, have been met [51].

### Statistical analysis

A statistical comparison was performed between the results obtained for the analysis of SAF in pharmaceutical dosage form using the proposed methods and those obtained using a previously reported HPLC method [10]. The calculated t and F-values are less than the tabulated values, indicating no statistically significant difference between the suggested and reported methods in terms of accuracy and precision, according to Supplementary Table S4.

## Conclusion

The primary objective of this study was to develop advanced and straightforward techniques, namely HPTLC-densitometry and HPLC–DAD, to simultaneously measure SAF and 4-HBD in the presence of SAF-forced degradation products. These methods were specifically designed for routine pharmaceutical analysis and stability testing. Thorough validation was conducted to ensure the reliability and effectiveness of the developed method, which was successfully employed to accurately determine and quantify SAF in both its pure powder form and pharmaceutical preparation. Notably, the following methods enabled the separation of SAF from its stress degradation products, impurities, and co-formulated excipients, without any significant interference while maintaining high accuracy and sensitivity. MS and IR methods were also used to identify the degradation products generated under various conditions. Furthermore, the developed HPLC–DAD method exploited its high sensitivity features to monitor the dissolution profiling of SAF, which was formulated as Parkimedine^®^ tablets in FDA-recommended media. The solubility of SAF in the FDA-recommended dissolution media was found to be high. However, for poorly soluble drugs, solubility can be improved by modifying the dissolution medium with surfactants and pH adjustments. Based on the results of the GAPI-Complex, AGREE, BAGI and the whiteness assessment, it is recommended that the proposed methods be used in the quality control laboratories for routine analysis of SAF because they are safe for the environment and the operator. Furthermore, the suggested techniques have the capacity to be utilized in forthcoming pharmacokinetic investigations of the mentioned drugs in various biological samples.

## Supplementary Information

Additional file1.

## Data Availability

All data generated or analysed during this study are included in this published article.
