# Supplementary material for: A novel stability-indicating chromatographic quantification of the antiparkinsonian drug safinamide in its pharmaceutical formulation employing HPTLC densitometry and ion-pair HPLC–DAD
Source: BMC Chem. 2024 Nov 1;18(1):212. doi: 10.1186/s13065-024-01315-y (PMC11529230; doi:10.1186/s13065-024-01315-y)
Supplement: Supplementary file 1 — Additional file1. [file 13065_2024_1315_MOESM1_ESM.docx]

**f)**

**e)**

**d)**

**c)**

**b)**

**a)**

**Thermal**

**R_f_ 0.53**

**Photolytic**

**R_f_ 0.53**

**Oxi Deg.**

**R_f_ 0.39**

**Alk Deg.**

**R_f_ 0.03**

**Acid Deg.**

**R_f_ 0.03**

**SAF**

**Rf 0.53**

**Fig. S1.** HPTLC-densitograms of (**a**) intact SAF; (**b**) acid hydrolysis in 5 N HCl refluxed for 5h; (**c**) alkali hydrolysis in 5 N NaOH refluxed for 5 h; (**d**) oxidative degradation in 30% H_2_O_2_ at room temperature for 24 h; (**e**) photolytic degradation for 6 h; and (**f**) thermal degradation in 110 °C oven for 5 h.

**Fig. S2. (a)** The IR spectrum of Safinamide intact drug.
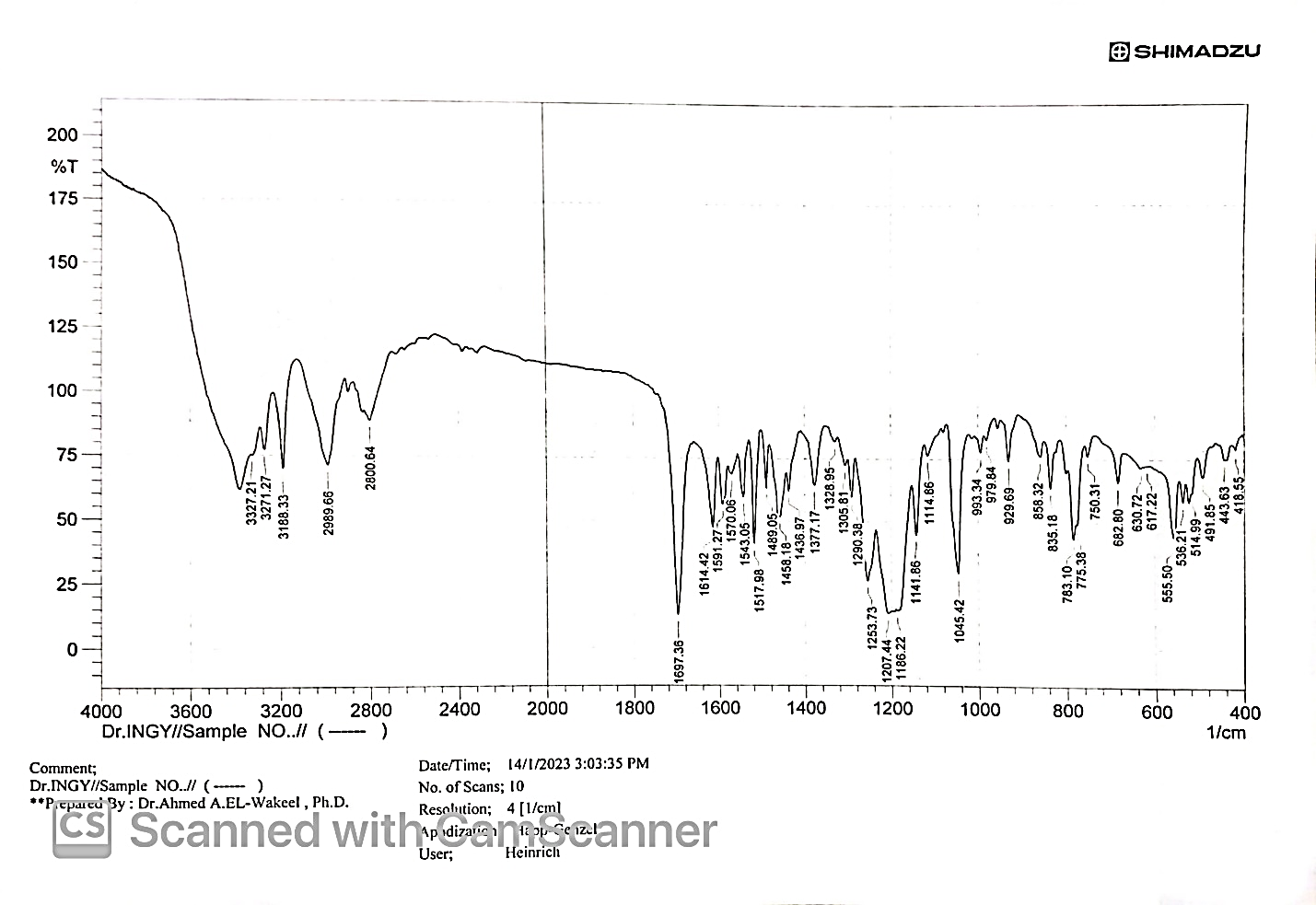


**a)**

**Amidic C=O**

**Forked** **NH_2_ band**

**Broad OH band**


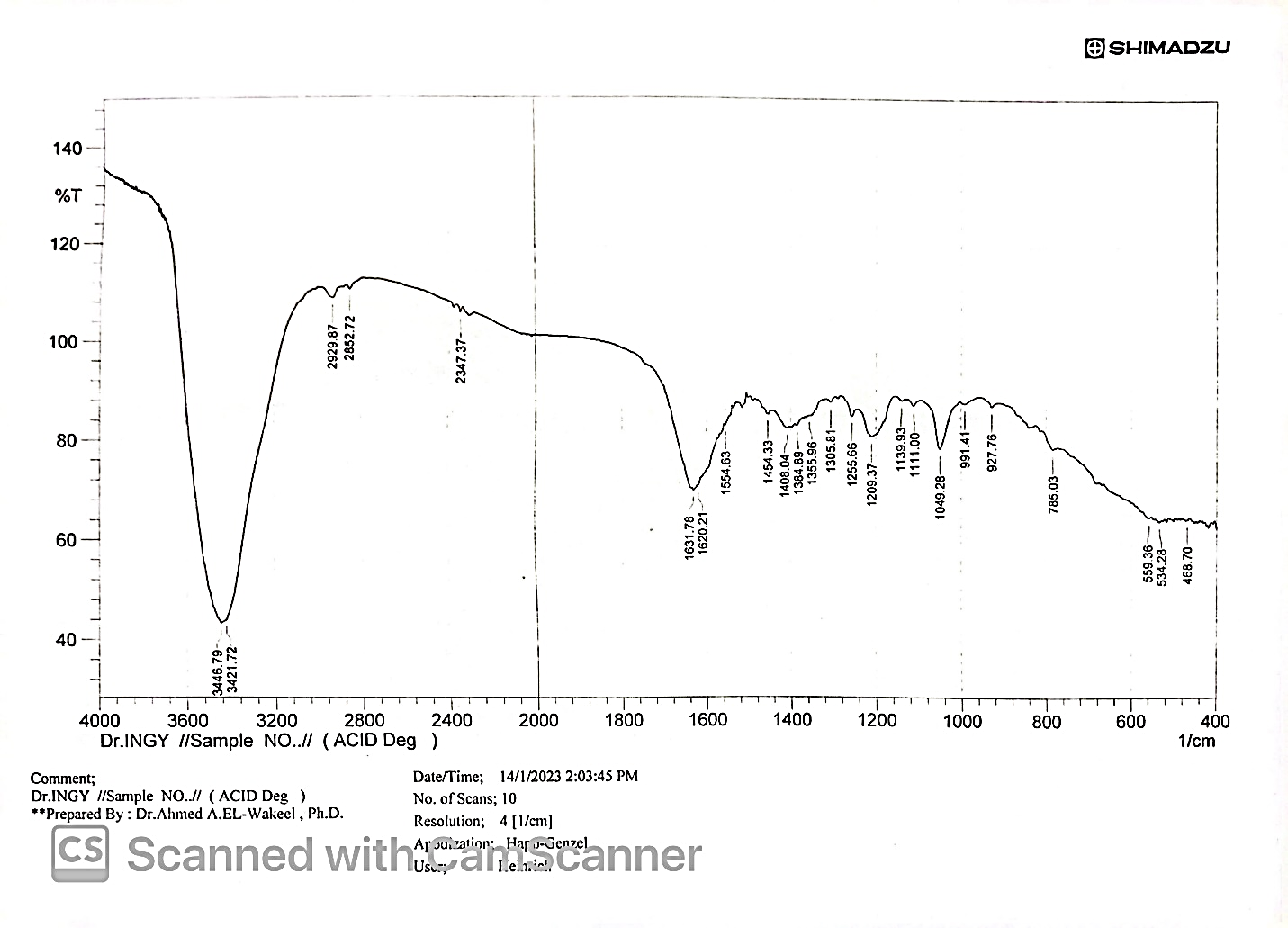


**b)**

**C=O band**

**Fig. S2. (b)** The IR spectrum of acidic degradation product of Safinamide.


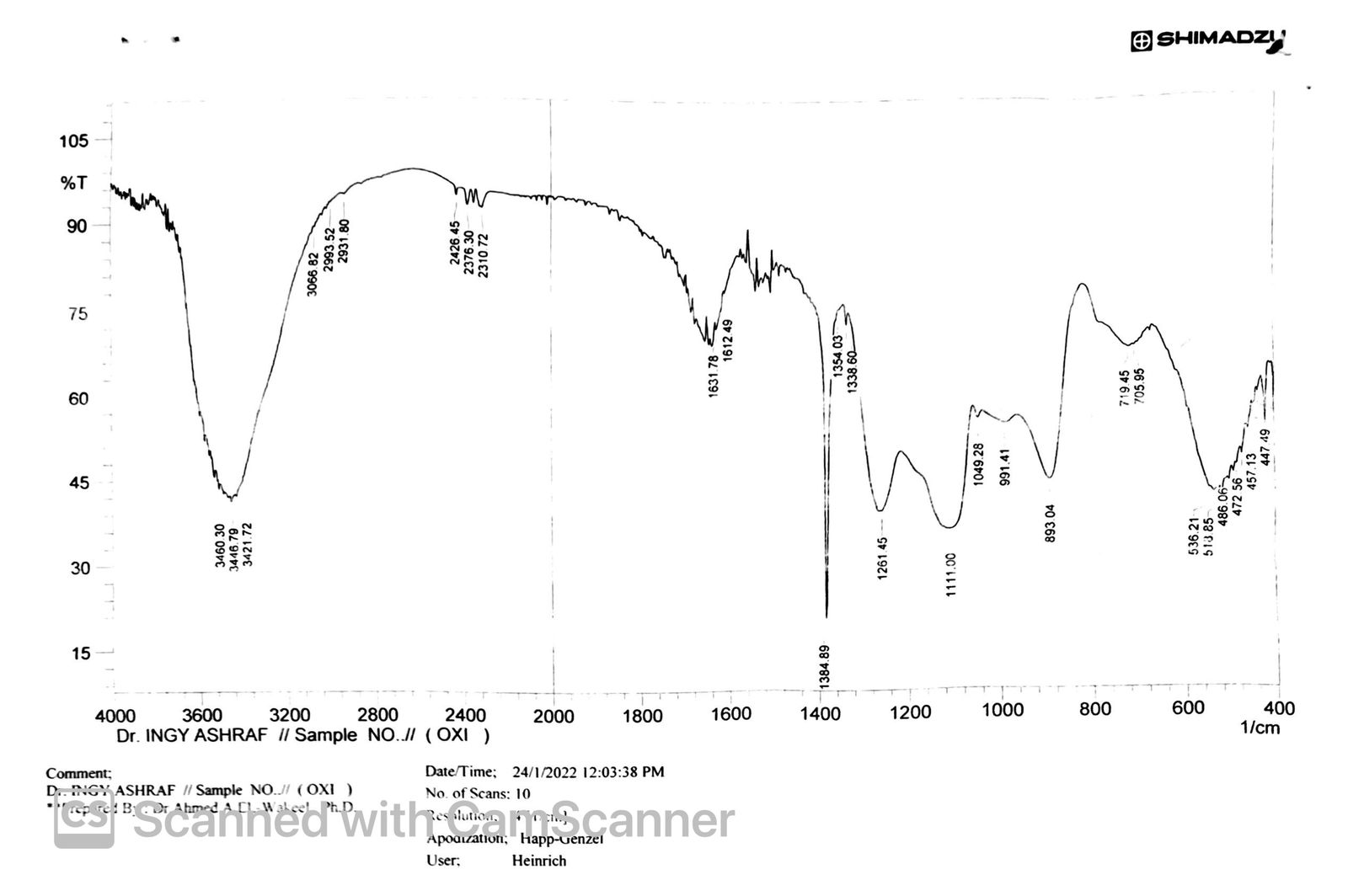


**c)**

**Broad OH band**

**C=O band**

**Fig. S2. (c)** The IR spectrum of basic degradation product of Safinamide.


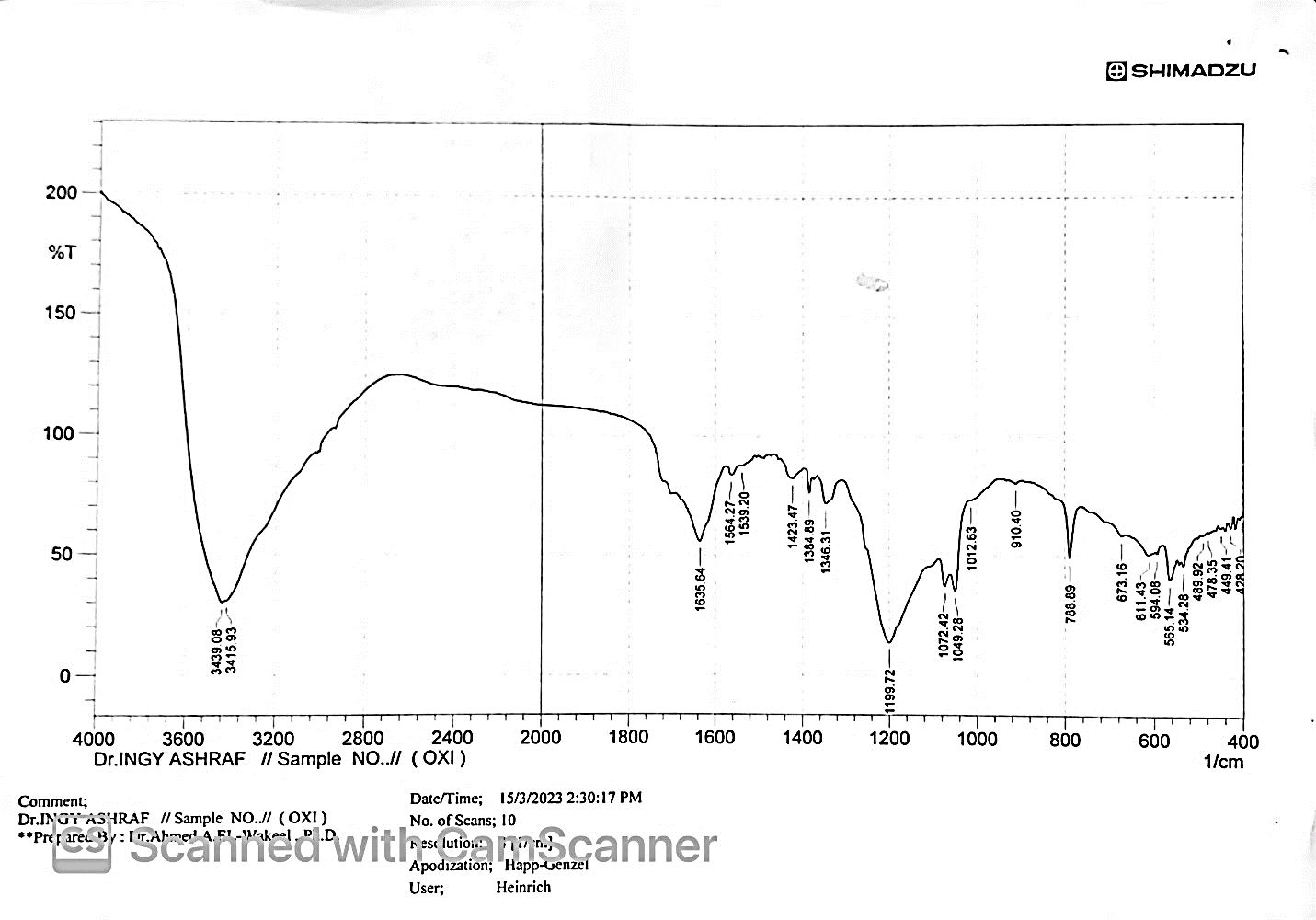


**d)**

**C=O band**

**Broad OH band**

**Forked NH_2_ band**

**Fig. S2. (d)** The IR spectrum of oxidative degradation product of Safinamide.


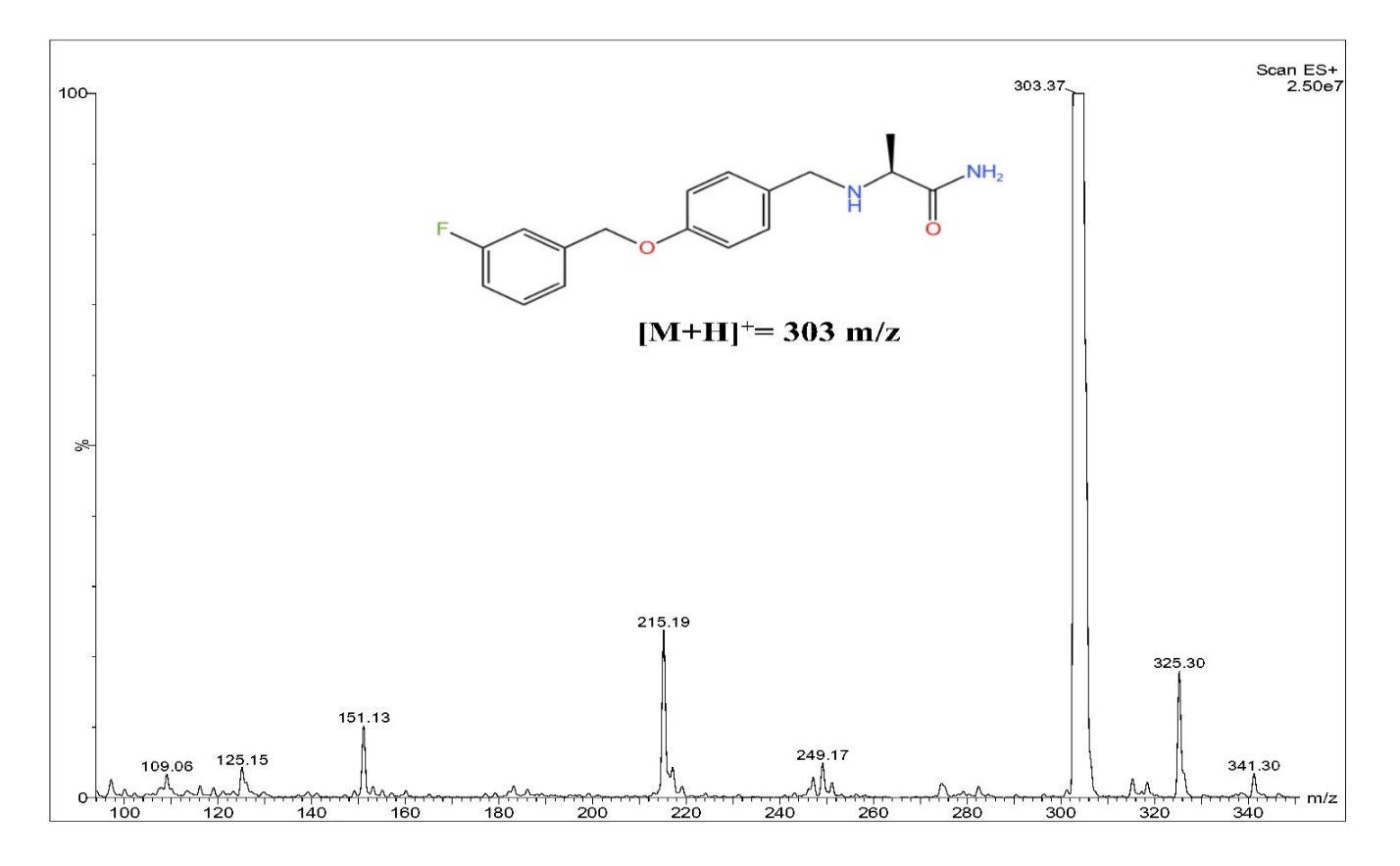
 **Fig. S3. (a)** The mass spectrum showing molecular ion peak at 303 m/z for Safinamide intact drug.

**a)**


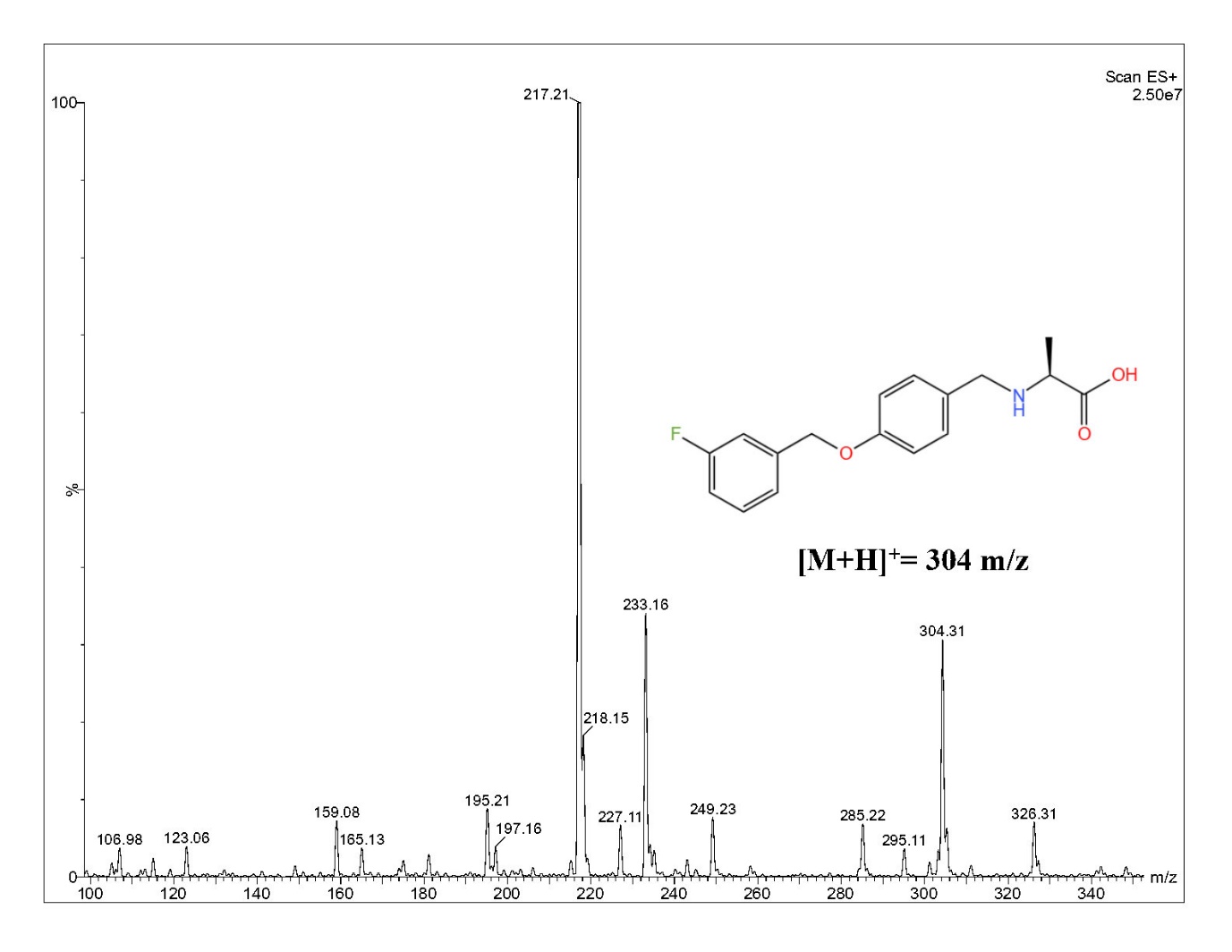


**b)**

**Fig. S3. (b)** The mass spectrum showing molecular ion peak at 304 m/z for acidic degradation product.

**
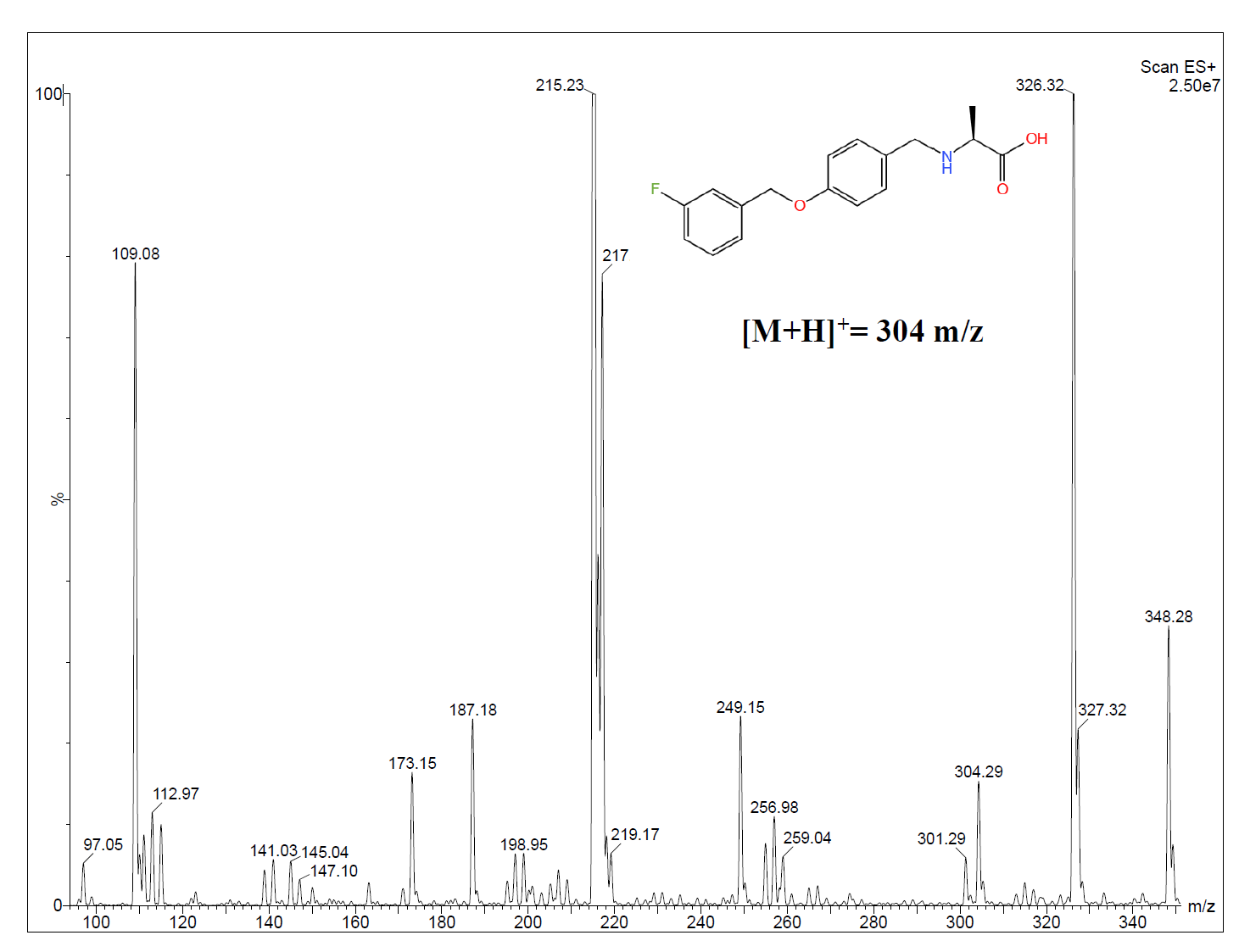
**

**c)**


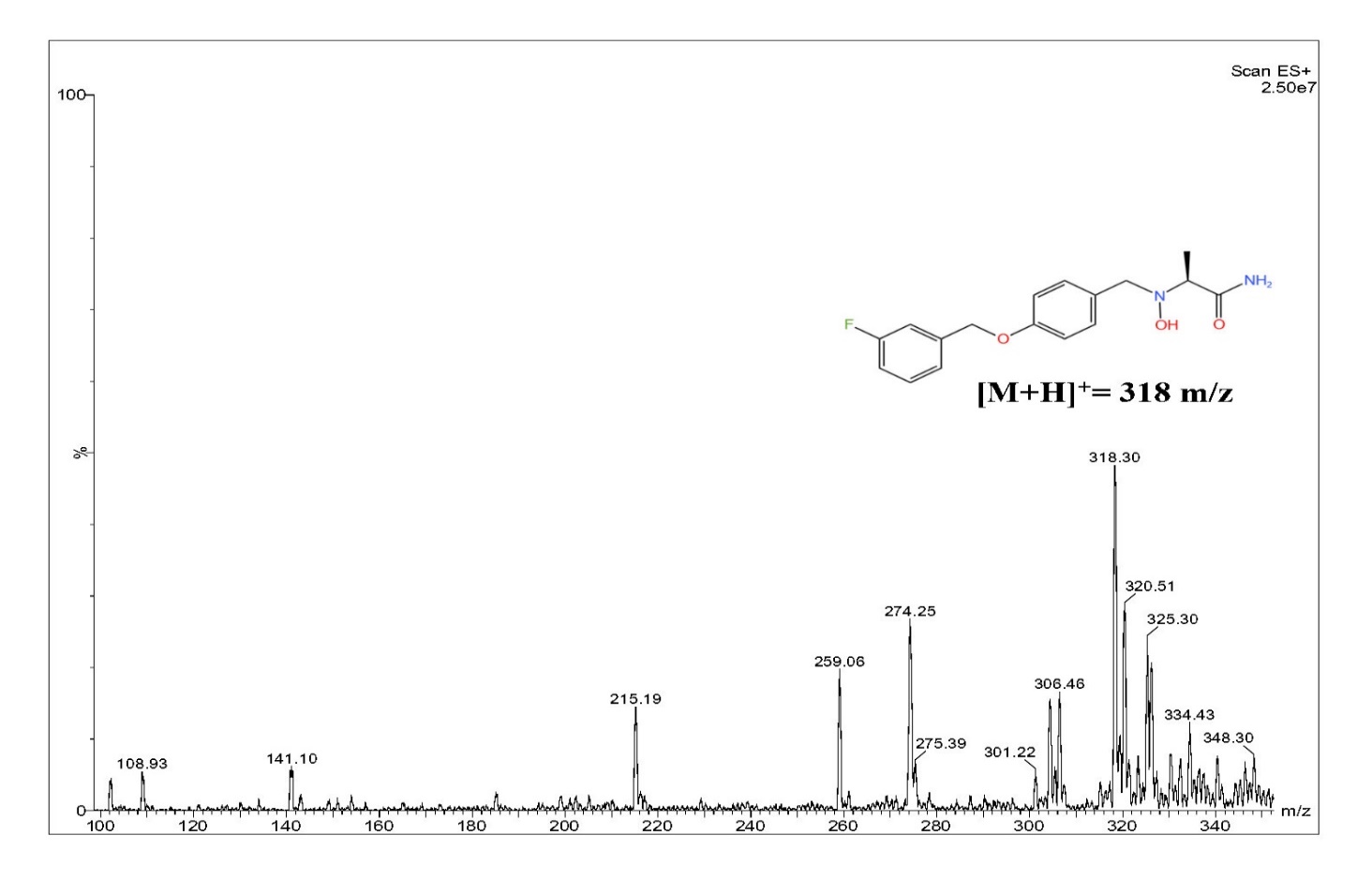
**Fig. S3. (c)** The mass spectrum showing molecular ion peak at 304 m/z for basic degradation product.

**d)**

**Fig. S3. (d)** The mass spectrum showing molecular ion peak at 318 m/z for oxidative degradation product.

| **Complex-GAPI pictogram** | | | |
| --- | --- | --- | --- |
| 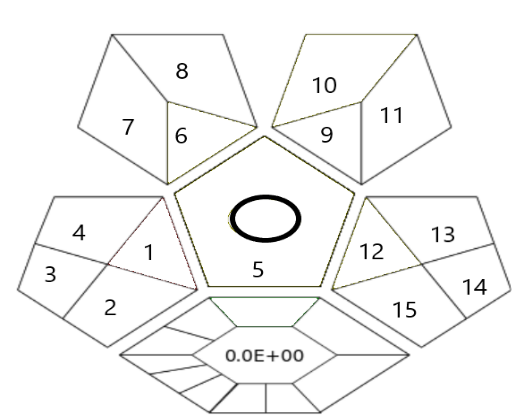 | | | |
| **Category** | | | |
| **Sample preparation** | **The proposed HPTLC method** | **The proposed HPLC method** | **The reported HPLC method** |
| 1. Collection | Off-line (Red) | Off-line (Red) | Off-line (Red) |
| 2. preservation | None (Green) | None (Green) | None (Green) |
| 3. Transport | Required (Yellow) | Required (Yellow) | Required (Yellow) |
| 4. Storage | None (Green) | None (Green) | None (Green) |
| 5. Type of method: direct or indirect | Simple procedures (Yellow) | Simple procedures (Yellow) | Simple procedures (Yellow) |
| 6. Scale of extraction | Microextraction (Yellow) | Microextraction (Yellow) | Microextraction (Yellow) |
| 7. Solvents/reagents used | Green solvents/reagents used (Yellow) | Green solvents/reagents used (Yellow) | Non-green solvents/reagents used (Red) |
| 8. Additional treatments | None (Green) | None (Green) | None (Green) |
| **Reagents and solvents** | | | |
| 9. Amount | <10 mL (Green) | 10–100 mL (Yellow) | 10–100 mL (Yellow) |
| 10. Health hazard | Moderately toxic (Yellow) | Moderately toxic (Yellow) | Moderately toxic (Yellow) |
| 11. Safety hazard | Special hazard is used (Yellow) | Special hazard is used (Yellow) | Special hazard is used (Yellow) |
| **Instrumentation** | | | |
| 12. Energy | ≤1.5 kW h per sample (Yellow) | ≤1.5 kW h per sample (Yellow) | ≤1.5 kW h per sample (Yellow) |
| 13. Occupational hazard | Emission of vapors to the atmosphere (Red) | Hermitization of the analytical process (Green) | Hermitization of the analytical process (Green) |
| 14. Waste | 1–10 mL (Yellow) | 1–10 mL (Yellow) | >10 mL (Red) |
| 15. Waste treatment | No treatment | No treatment | No treatment |
| **Method type (Oval in the middle of GAPI)** | | | |
| Type of analysis | qualitative and quantitative | qualitative and quantitative | qualitative and quantitative |

**Table S1.** Complex-GAPI evaluation for the proposed methods.

**Table S2.** AGREE evaluation reports for the proposed HPTLC-densitometric, HPLC-DAD methods, and the reported one.


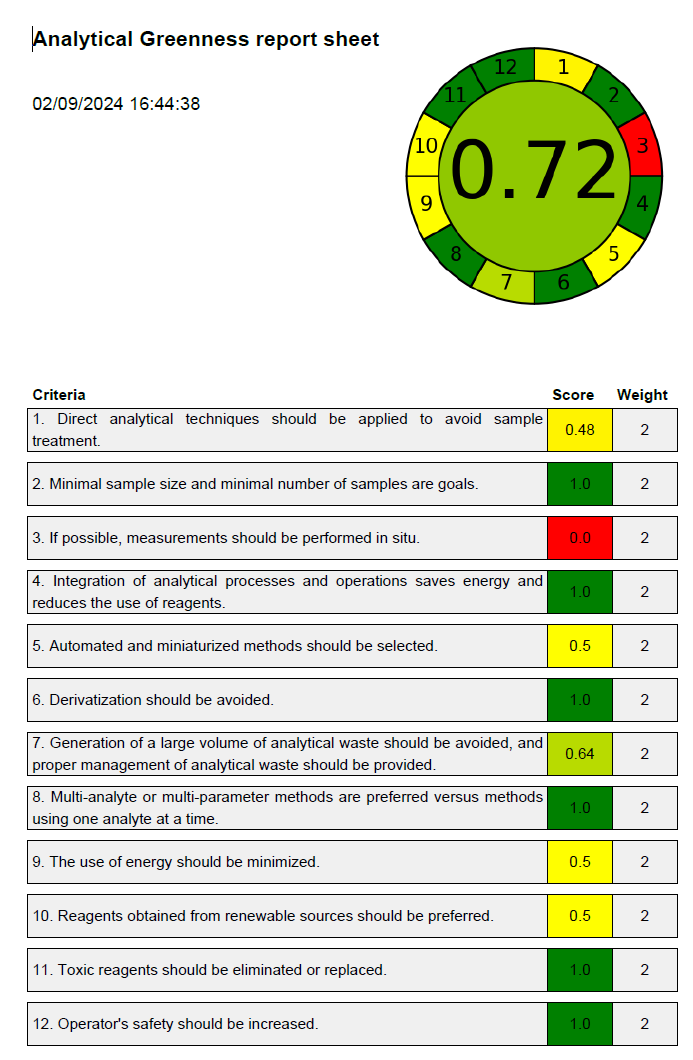


**For proposed HPTLC-densitometric method**


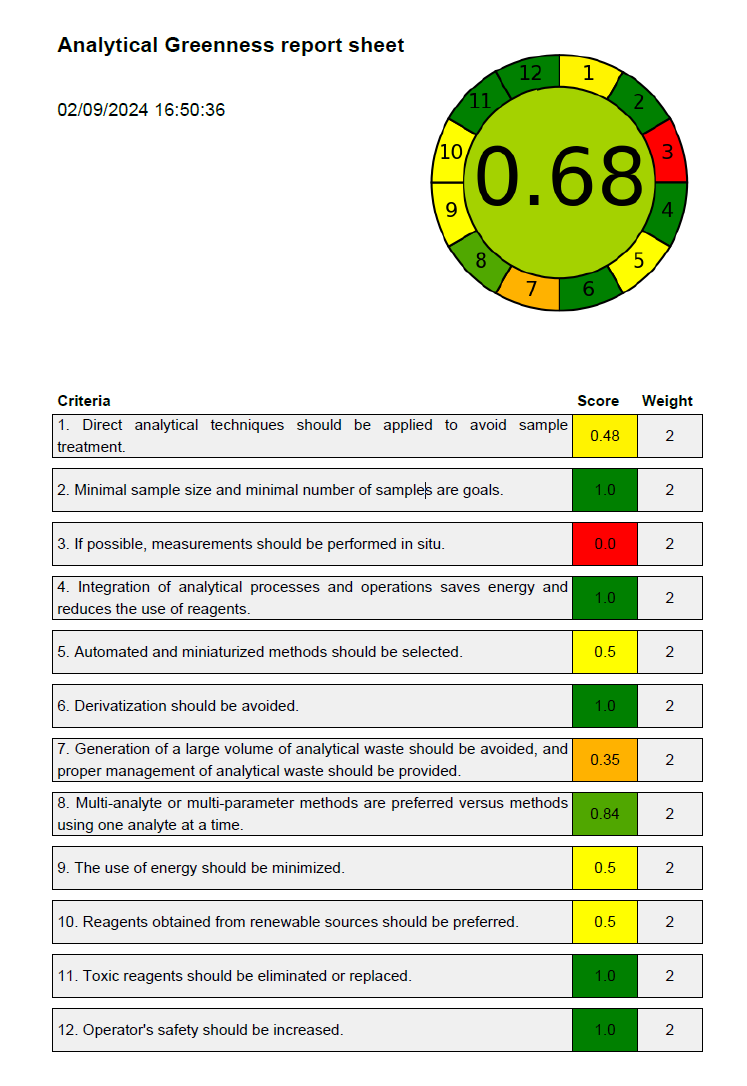


**For proposed HPLC-DAD method**


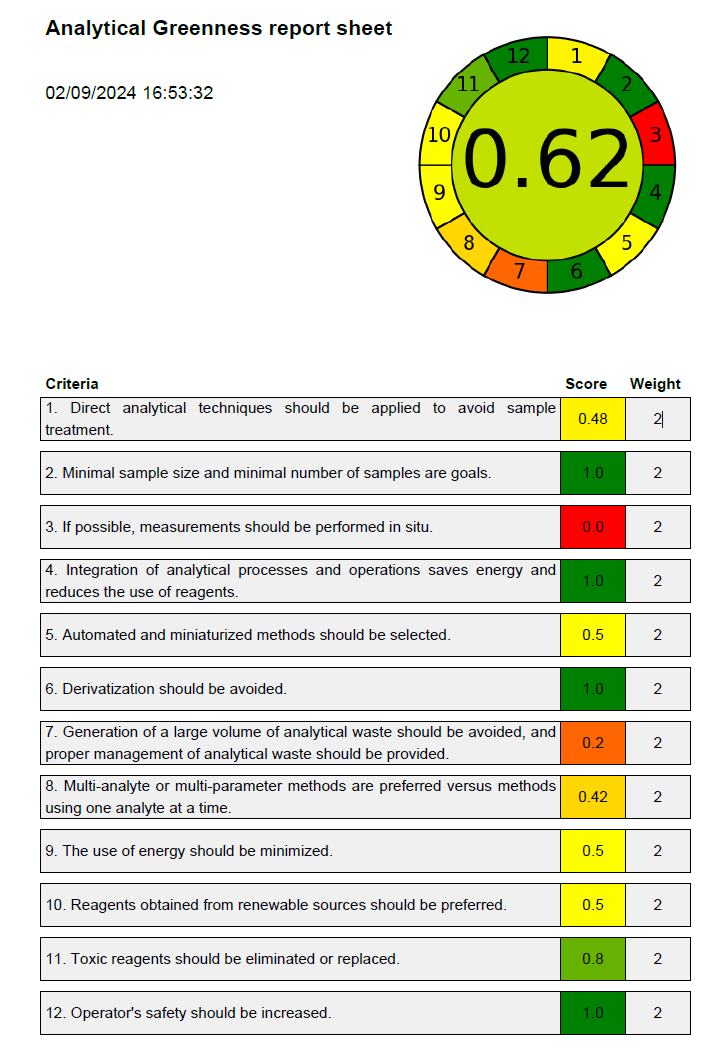


**For reported HPLC-UV method**

**Table S3.** Whiteness assessment for the proposed HPTLC, HPLC-DAD, and the reported method.

**Table S4.** Statistical comparison of the results obtained by the proposed HPTLC-densitometry and HPLC-DAD methods and the reported method for Safinamide mesylate in pharmaceutical dosage form.

| Parameters | HPTLC-densitometry | HPLC-DAD method | Reported method ^a)^ |
| --- | --- | --- | --- |
|  | **SAF** | **SAF** | **SAF** |
| Mean | 100.13 | 99.63 | 99.74 |
| SD | 1.815 | 0.523 | 1.013 |
| n | 5 | 5 | 5 |
| Variance | 3.294 | 0.274 | 1.026 |
| Student’s t-test (2.306) ^b)^ | 0.42 | 0.22 | ------- |
| F value (6.39) ^b)^ | 3.21 | 3.74 | ------- |

1. HPLC method using Inertsil ODS-3 column (250 × 4.6 mm, 5 μm) as analytical column, mobile phase consists of a mixture of 0.1% formic acid in water (pH adjusted to 5.0) and acetonitrile as the mobile phase, the flow rate was 1.0 mL/min, and detected at 220.0 nm [10].
2. Tabulated t- and F values at P = 0.05.
